# NAT10 induces N4-acetylcytidine modification of AdipoR1-mediated mitochondrial biogenesis against endothelial-to-mesenchymal transition in hypertension

**DOI:** 10.1186/s10020-025-01321-3

**Published:** 2025-11-18

**Authors:** Huichao Pan, Lei Song, Zeyi Cheng, Jie Zhu, Jun Zhou, Zhongqing Xu, Min Zhang

**Affiliations:** 1 Institute of Cardiovascular Diseases, Division of Cardiology, Tongren Hospital, Shanghai Jiao Tong University School of Medicine, Shanghai, 200336 China; 2 Department of Cardiovascular Surgery, Ruijin Hospital, Shanghai Jiao Tong University School of Medicine, Shanghai, China; 3 Center for Translational Neurodegeneration and Regenerative Therapy, Tenth People’s Hospital of Tongji University, Shanghai, China; 4 Department of General Practice, School of Medicine, Tongren Hospital, Shanghai Jiao Tong University, Shanghai, China

**Keywords:** Hypertension, Endothelial-to-mesenchymal transition, Endothelial cells, NAT10, Ac4C, AdipoR1, Mitochondrial biogenesis

## Abstract

**Background:**

Endothelial-to-mesenchymal transition (EndMT) in endothelial dysfunction exacerbates hypertension. However, the regulatory mechanisms underlying EndMT in hypertension are yet to be elucidated.

**Methods:**

The N-acetyltransferase 10 (NAT10) and N4-acetylcytidine (ac4C) levels were determined in hypertensive mice, spontaneously hypertensive rats (SHRs), and angiotensin II (Ang II)-treated human umbilical vein endothelial cells (HUVECs). Biological functional assays were performed with lentiviral vectors to induce the overexpression or knockdown of NAT10 in vivo and in vitro. The detailed mechanisms underlying the role of ac4C-mediated posttranscriptional regulation in hypertension were investigated by combining ac4C-RIP-seq with RNA-seq, RIP-qRCR, mRNA stability, and dual-luciferase assays. Mitochondrial biogenesis and function were assessed via reactive oxygen species (ROS) and mitochondrial ROS (mtROS) staining; estimation of ATP levels, the mitochondrial membrane potential (MMP), and the mtDNA content; and evaluation of mitochondrial respiratory chain complex activities.

**Results:**

The results revealed that NAT10 and ac4C levels are higher in the hypertensive mice descending thoracic aorta tissues, SHRs descending thoracic aorta samples, and Ang II-treated HUVECs compared to the control groups. NAT10 overexpression inhibits EndMT in hypertension, which is partly due to the inhibition of endothelial dysfunction, whereas NAT10 inhibition has the opposite effect. Mechanistically, NAT10 inhibited endothelial dysfunction in hypertension through increased AdipoR1 mRNA ac4C acetylation. Moreover, NAT10 induced AdipoR1 expression, leading to increased mitochondrial biogenesis and function in Ang II-treated ECs via p38 MAPK/PGC-1α signaling.

**Conclusions:**

The current data highlighted the molecular mechanisms of NAT10-induced ac4C acetylation and implied that the NAT10-AdipoR1 axis might be the therapeutic target to inhibit endothelial dysfunction and EndMT in hypertension.

**Supplementary Information:**

The online version contains supplementary material available at 10.1186/s10020-025-01321-3.

## Introduction

Hypertension is a leading cause of cardiovascular diseases (Boutouyrie et al. 2021; Jin et al. 2023; Mills et al. 2020). Although pharmacological therapies have been administered to inhibit hypertension, the current treatments are only partially effective (Kitagawa 2022; Addison et al. 2023; Moick et al. 2023). Therefore, novel mechanisms and treatment targets are urgent requisites to inhibit the development of hypertension.

Increasing evidence has shown that vascular endothelial dysfunction indicates the onset of hypertension. Endothelial heterogeneity and plasticity in hypertensive vasculature imply that hypertension is a complex condition. Hypertension induces endothelial-to-mesenchymal transition (EndMT), a cellular process involving the transdifferentiation of ECs to mesenchymal or myofibroblastic phenotype, which accumulates in the vascular injury site (Kovacic et al. 2019). During the EndMT process, ECs lose the endothelial phenotype to obtain mesenchymal or myofibroblastic characteristics; thus, the expression of endothelial markers (such as CD31 and VE-cadherin) decreases, and that of EndMT markers (such as N-cadherin and SM22α) increases. However, the complex mechanism that regulates EndMT in hypertension is still unknown. Interestingly, epigenetic alterations, such as DNA methylation and N6-methyladenosine modification, are associated with EndMT in hypertension (Wang et al. 2024a ; Ray et al. 2024). METTL3 deficiency triggers EndMT and vascular remodeling in pulmonary hypertension (Kang et al. 2024). In addition, N4-acetylcytidine (ac4C) is a common epigenetic modification of RNAs, including messenger RNAs (mRNAs) and noncoding RNAs (ncRNAs) (Zhang et al. 2024a). N-acetyltransferase 10 (NAT10), the ac4C “writer” protein, alters mRNA stability and translation through remodeling ac4C acetylation (Xie et al. 2023). Abnormal ac4C modification is associated with cancer and cardiovascular diseases (Xu et al. 2023; Liao et al. 2023; Wang et al. 2023; Zhang et al. 2024b). NAT10 promotes collagen deposition and cardiac systolic dysfunction through ac4C modification of *Amotl1* mRNA during myocardial infarction injury (Wang et al. ,2024 b). A recent study revealed that the levels of both NAT10 and ac4C modifications increased in damaged human and rodent arteries. Moreover, NAT10 knockdown inhibited vascular smooth muscle cell (VSMC) phenotype switching and neointima formation (Yu et al. 2024). Despite these advancements, the relationships among NAT10, endothelial dysfunction, and EndMT in hypertension have not yet been reported.

Adiponectin (APN) is an adipocyte-specific protein involved in cardiovascular function. The plasma APN level is related to endothelial dysfunction in various stages of cardiovascular disease in patients (Njajou et al. 2009). Moreover, inhibition of APN receptor (AdipoR1 and AdipoR2) expression causes vascular senescence and atherosclerosis (Hafiane 2024). Conversely, activated AdipoR1 enhances glucose and free fatty acid consumption, inhibits the inflammatory response, and accelerates mitochondrial biogenesis (Song et al. 2023). Additionally, mitochondrial biogenesis is essential for regulating the structure and function of organelles. Peroxisome proliferator-activated receptor-gamma coactivator α (PGC-1α), a key target of AdipoR1, mediates mitochondrial biogenesis (Iwabu et al. 2010). Endothelial cell-specific knockdown of PGC-1α decreases angiogenesis and exacerbates pulmonary hypertension (Fujiwara et al. 2023). Intriguingly, PGC-1α inhibition aggravates EndMT in pulmonary hypertension through an assault on endothelium integrity (Cai and Chen 2022). Thus, elucidating the pathological mechanism of the AdipoR1/PGC-1α axis may provide a novel therapy against endothelial dysfunction and EndMT in hypertension.

Herein, we explored the effect of NAT10-induced ac4C acetylation on endothelial dysfunction and EndMT in hypertension and clarified the mechanism through which the NAT10/ac4C/AdipoR1/PGC-1α axis inhibits endothelial dysfunction via the regulation of mitochondrial biogenesis and function. Taken together, our data highlighted that increasing NAT10-induced ac4C acetylation might be an effective therapeutic approach for hypertension.

## Materials and methods

### Animals

Wild-type (WT) C57BL/6 mice (20–25 g, Male, 8 weeks old), Wistar-Kyoto rats (WKYs, Male, 10 weeks old), and spontaneously hypertensive rats (SHRs, Male, 10 weeks old) were obtained from Vital River Laboratory Animal Technology Co. Ltd. (Beijing, China). All animals were Maintained in a pathogen-free facility on a 12-h light-dark cycle with food and water ad libitum. The animal experimental protocols were approved by Shanghai Tongren Hospital (2023-104-01). Animal experiments were performed according to the principles of laboratory animal care and Chinese national laws and the guidelines supplied by the National Institutes of Health (NIH) Guide for the Care and Use of Laboratory Animals. The work has been reported in accordance with the ARRIVE guidelines (Animals in Research: Reporting In Vivo Experiments).

### Hypertensive mice and administration

A hypertensive mouse model was established by administering angiotensin II (Ang II) via an osmotic pump. The C57BL/6 mice were anesthetized and implanted with an osmotic pump (1004, Alzet, USA). Ang II (490 ng/kg/min) or normal saline was administered to the animals for 4 weeks (Yin et al. 2022).

Experiment 1: To evaluate the effects of NAT10 overexpression on Ang II-induced hypertension, the mice were grouped and treated as follows: OE-NC group: the mice were injected with the control lentivirus (OE-NC, 1 × 10^8^ IU/mL, 100 µL) via the tail vein (*n* = 6); OE-NAT10 group: the mice were administered the overexpressing NAT10 lentivirus (OE-NAT10, 1 × 10^8^ IU/mL, 100 µL) via the tail vein (*n* = 6). Four weeks after Ang II (490 ng/kg/min) administration, all the animals were euthanized via terminal anesthesia with 4% isoflurane, and the descending thoracic aorta tissues that were stored until further use were excised.

Experiment 2: To evaluate the effect of NAT10 inhibition on Ang II-induced hypertension, the mice were grouped and treated as follows: sh-NC group: the mice were injected with the control lentivirus (sh-NC, 1 × 10^8^ IU/mL, 100 µL) via the tail vein (*n* = 6); sh-NAT10 group: the mice were administered sh-NAT10 lentivirus (sh-NAT10, 1 × 10^8^ IU/mL, 100 µL) via the tail vein (*n* = 6). The sequences of the shRNAs used are listed in the Additional file Material 6 : Table s1 . Four weeks after Ang II (490 ng/kg/min) administration, all the animals were euthanized via terminal anesthesia with 4% isoflurane, and the descending thoracic aorta tissues that were stored until further use were excised.

Experiment 3: To evaluate the effects of the NAT10 inhibitor remodelin on Ang II-induced hypertension, the mice were grouped and treated as follows: DMSO group: mice with dimethylsulfoxide (DMSO) and Ang II treatment (*n* = 6). The mice were treated with DMSO daily by intraperitoneal injection for 7 consecutive days and then treated with Ang II (490 ng/kg/min) for 4 weeks . Remodelin group: mice subjected to remodeling and Ang II treatment (*n* = 6). The mice were treated with remodelin daily by intraperitoneal injection for 7 consecutive days at 25 mg/kg/day (Ma et al. 2024) and then treated with Ang II (490 ng/kg/min) for 4 weeks. The average body of mice (20–25 g) did not change significantly throughout the experiment (Fig. S1). After treatment, all the animals were euthanized via terminal anesthesia with 4% isoflurane, and the descending thoracic aorta tissues were excised and stored until further use.

### Blood pressure

Blood pressure was detected by the tail-cuff method, and the average value was determined as described previously.

### Histopathological analysis

The descending thoracic aorta tissues were fixed in 4% formaldehyde, embedded in paraffin, and sliced into 5-µm serial sections. These sections were stained using hematoxylin-eosin (G1120, Solarbio Science & Technology Co., Ltd, China) and Masson’s staining kits (G1340, Solarbio Science & Technology Co., Ltd, China), respectively.

### Immunofluorescence (IF) staining

The descending thoracic aortic sections were blocked with blocking buffer (3% bovine serum albumin in PBS) for 60 min and stained with primary antibodies containing NAT10 (Abcam, ab194297, rabbit, 1:500), CD31 (MCE, YA806, mouse, 1:100) and SM22α (Proteintech, 10493-1-AP, rabbit, 1:100) overnight at 4 °C and the corresponding Alexa-conjugated secondary antibody containing Donkey Anti-Mouse IgG H&L (Alexa Fluor^®^ 488) (Abcam, ab150105, 1:500), Goat Anti-Rabbit IgG H&L (Alexa Fluor^®^ 594) (Abcam, ab150080, 1:1000), Donkey Anti-Mouse IgG H&L (Alexa Fluor^®^ 594) (Abcam, ab150108, 1:500), Goat Anti-Rabbit IgG H&L (Alexa Fluor^®^ 488) (Abcam, ab150077, 1:1000) at room temperature for 1 h. The nuclei were counterstained with DAPI (C0065, Solarbio) for 5 min, and the fluorescence images were captured under a fluorescence microscope (Olympus). The negative control was used and the section was stained with nonimmune IgG and then treated with the same secondary antibodies, and no specific staining was captured. Three visual fields were captured randomly from every sample and analyzed using ImageJ software as described previously (Zhang et al. 2022).

### Cell culture and treatment

Human umbilical vein endothelial cells (HUVECs, DFSC-EC-01, Sciencell, Shanghai, China) were cultured in the complete growth medium (PCM-H-040, Sciencell, Shanghai, China) at 37 °C as described previously. HUVECs from passages 3–5 was utilized for all experiments. In order to mimic the pathological changes of hypertension in vitro, Ang II (1 µM, Sigma, USA) was added to HUVECs for 24 h. Moreover, remodelin (40 µM, Selleck, USA) was solubilized in DMSO before being added to HUVECs (Qu et al. 2024), and HUVECs were pretreated with remodelin for 60 min.

### Endothelial cell transfection

For the gain-of-function assay, the full-length NAT10 gene was cloned and inserted into the PGMLV-CMV-MCS-3×Flag-PGK-Puro vector (Genomeditech). HUVECs (1 × 10^5^) were incubated in six-well plates at 37 °C for 24 h until they reached 80% confluency. HUVECs were transfected with the lentiviral vector (multiplicity of infection (MOI) = 20) using Lipofectamine 3000 (Thermo Fisher Scientific, L3000008) for 48 h, and puromycin (2 µg/mL) was added to the medium of the transduced cells. The transfection efficiency was determined using western blotting. For the loss-of-function assay, HUVECs (1 × 10^5^) were incubated in six-well plates at 37 °C for 24 h until they reached 80% confluency. HUVECs were either transfected with shRNA lentivirus targeting NAT10 (sh-NAT10) or control shRNA (sh-NC, MOI = 20), which were cloned and inserted into the PGMLV-SB3 vector (Genomeditech) via Lipofectamine 3000 (Thermo Fisher Scientific, L3000008) for 48 h. The transfection efficiency was determined by western blotting. Sequences of shRNA were listed in the Additional file Material 6 : Table s1 .

### EC function assay

EC viability was measured using the cell counting kit (CCK)−8 kit (CK04, Dojindo, China). The migration ability of the ECs was detected using Transwell assay (8 μm, BD Biosciences, USA). HUVECs (5 × 10^4^) were added into the upper chamber, and DMEM medium with 20% FBS was added to the lower chamber. The plates were incubated at 37 °C for 24 h, and the migrated cells were labeled with Calcein M for 15 min. The number of migrated ECs was detected by a fluorescence microscope. The tube formation ability was measured by Matrigel assay (356231, Corning, USA). Firstly, Matrigel (70 µL/well) was added to a 96-well culture plate at 37 °C for 1 h. HUVECs (1 × 10^4^ cells/100 µL) were added into the well at 37 °C for 24 h, and tube formation was detected by an inverted optical microscope. The tubule images were acquired and analyzed by the Angiogenesis Analyzer plug-in of ImageJ, which were measured by the number of Nb master junction per field as described previously (Zhang et al. 2020; Liu et al. 2024).

### ac4C RNA Immunoprecipitation sequencing (ac4C-RIP-seq)

Total RNA was obtained by TRIzol (Invitrogen, CA, USA), and small RNAs were harvested using the RNA Clean and Concentrator-25 kit (R1017, ZYMO Research, USA). Next, the small RNAs were treated with the anti-ac4C antibody (Abcam, ab252215, 1:50). The input and immunoprecipitated small RNAs were incubated with AlkB and AlkB-D135S, and the cDNA library were constructed using Multiplex Small RNA Library Prep Set for Illumina kit (New England Biolabs, USA) and sequenced on the Illumina NovaSeq 6000 platform.

### RNA-seq and pathway enrichment analysis

Total RNA was harvested, RNA quality was checked by determining the A260/A280 ratios, and the RNA concentration was quantified. Qualified RNA was used for RNA-seq, and the RNA libraries were constructed (Next Ultra II Directional RNA Library Prep Kit, New England Biolabs) and sequenced. After quality control, the differentially expressed genes (DEGs) were analyzed using the DESeq2 R package. The thresholds for DEGs were a *p-*value < 0.05 and a fold change (FC) ≥ 2. Functional gene ontology (GO) and Kyoto Encyclopedia of Genes and Genomes (KEGG) enrichment analyses were subsequently performed.

### RIP-qPCR

HUVECs were lysed using an immunoprecipitation lysis buffer (Magna RIP kit, 17–700, Millipore, USA). The magnetic beads were incubated with anti-ac4C (1:100, ab252215, Abcam), NAT10 (1:100, ab194297, Abcam), or IgG (1:100, ab172730; Abcam) antibodies at 4 ℃. The RNA was harvested from the eluted bound complexes and analyzed by qRT-PCR.

### mRNA stability assay

HUVECs were incubated with actinomycin D (5 µg/mL, Sigma) for 6 h, following which the half-life of mRNA was analyzed by qRT-PCR.

### Detection of reactive oxygen species (ROS), mitochondria ROS (mtROS), ATP level, mitochondrial membrane potential (MMP), mtDNA content, and mitochondrial respiratory chain complex activities

The levels of ROS and mtROS were quantified using the 2’,7’-dichlorodihydrofluorescein diacetate (H2DCF-DA) kit (S0035S, Beyotime, China) and MitoSO™ Red Mitochondrial Superoxide Indicator kit (S0061M, Beyotime, China). The ATP level was detected using the ATP assay kit (S0027, Beyotime, China), and MMP was evaluated using the JC-1 probe staining kit (C2003S, Beyotime, China). The mtDNA content was quantified by qPCR, and the activities of mitochondrial respiratory chain complex I, II, III, IV, and V were assessed using commercial assay kits (Solarbio Science & Technology Co., Ltd, China) (Zhuang et al. 2024).

### qRT-PCR and Western blot

Total RNA was obtained by TRIzol reagent (Invitrogen, USA) and reverse-transcribed into cDNA (RR037A, TaKaRa, Dalian, China). Then, qRT-PCR was performed using the TB Green^®^ Premix Ex Taq™ II FAST qPCR kit (CN830A, TaKaRa, Dalian, China). The qRT-PCR results were analyzed by the 2^−ΔΔCT^ method. The primer sequences are listed in the Additional file Material 6 : Table s1 . For western blotting, total protein was extracted with a ProteoPrep^®^ total protein isolation kit (Sigma, MO, USA), and the protein concentration was measured with a BCA kit (Beyotime, Shanghai, China). The samples (40 µg) were separated via electrophoresis and transferred to polyvinylidene fluoride (PVDF) membranes. After blocking with 5% non-fat milk in TBST for 60 min, the membranes were blotted with the following primary antibodies: NAT10 (Proteintech, 133651-AP, rabbit, 1:1000), CD31 (Abcam, ab281583, rabbit, 1:1000), VE-cadherin (Abcam, ab33168, rabbit, 1:1000), N-cadherin (Abcam, ab33168, rabbit, 1:5000), SM22α (Proteintech, 104931-AP, rabbit, 1:1000), collagen I (Abcam, ab316222, rabbit, 1:1000), collagen III (Abcam, ab184993, rabbit, 1:1000) and GAPDH (Proteintech, 10494-1-AP, rabbit, 1:10000), and the membranes were incubated with secondary antibodies (1:5000, ab288151, Abcam) for 1 h. The immunoreactive bands were detected on a chemiluminescence imager (Bio-Rad)(Wang et al. 2024c).

### Measurement of ac4C level by liquid chromatography with tandem mass spectrometry (LC–MS/MS)

The mRNA (1 µg) was added to buffer (S1 nuclease, alkaline phosphatase, and phosphodiesterase) at 37 °C. After complete digestion, the mixture was extracted with chloroform, and the aqueous layer was harvested for LC-ESI-MS/MS analysis (AB SCIEX QTRAP 6500+). The ac4C levels were calculated from a standard curve generated from pure nucleoside standards.

### Dual-luciferase reporter assay

The 3’UTR of wild-type (WT) AdipoR1 was inserted into the pGL3 luciferase plasmid (Promega, USA). The AdipoR1 3′-UTR mutation (Mut) was obtained via a QuikChange Multiple Site-Directed Mutagenesis Kit (Stratagene) and inserted into pGL3 luciferase plasmids (Promega, USA). Then, the plasmids were transfected into cells with Lipofectamine 3000 agent (Invitrogen) for 48 h. Luciferase activity was detected with the Dual Luciferase Reporter Assay Kit (E1910, Promega, USA).

### Statistical analysis

All data are expressed as the mean ± standard deviation (SD) from at least three independent experiments obtained using GraphPad Prism 8.0. The Shapiro-Wilk test was used to analyze data normality. Statistical analysis was performed using a t-test (unpaired, two-sided) between two groups or one-way ANOVA followed by Tukey’s multiple comparisons test for multiple-group comparisons, and *p* < 0.05 was considered statistically significant.

## Results

### High levels of ac4C and NAT10 were associated with hypertension

**Fig. 1:**
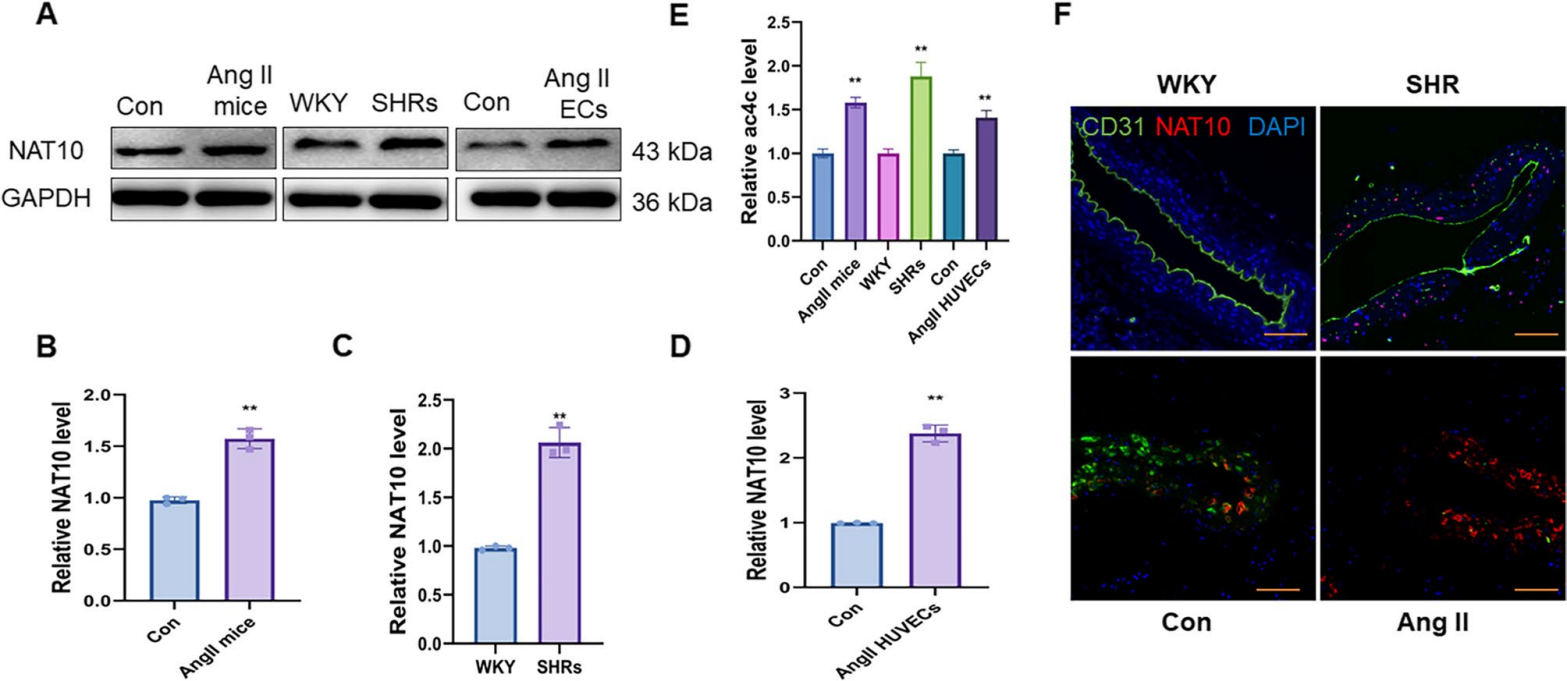
High NAT10 and ac4C levels in hypertension groups compared to the control groups. WB assay and the quantitative analysis of NAT10 level in hypertensive mice descending thoracic aortic tissues (**A**, **B**; *n* = 3**)**, SHRs descending thoracic aortic samples (**A**, **C**; *n* = 3**)** and Ang II treated HUVECs (**A**, **D**; *n* = 3**)**. **(E)** The ac4C level in hypertensive mice descending thoracic aortic tissues, SHRs descending thoracic aortic samples and Ang II treated HUVECs (*n* = 3). **(F)** Representative IF staining of NAT10 and CD31 in hypertensive mice descending thoracic aortic tissues, SHRs descending thoracic aortic samples and the control tissues. Fluorescence in green represents CD31, while fluorescence in red represents NAT10 and fluorescence in blue represents DAPI. Scale bar, 100 μm. (representative images; *n* = 6). Data are presented as mean ± SD. **p < 0.01. Statistical tests were performed using unpaired two-tailed Student’s t-test

To investigate whether ac4C and its related enzymes mediate hypertension, both ac4C and NAT10 levels were evaluated. In the present study, we used two hypertensive animal models: C57BL/6 mice treated with Ang II (490 ng/kg/min) for 4 weeks and SHRs. We found that Ang II increases systolic blood pressure (SBP) in C57BL/6 mice (Fig. S2A), and SHRs exhibit higher SBP compared to the WKY group (Fig. S2B). Compared to the respective control groups, the levels of NAT10 (Fig. 1A–D) and ac4C (Fig. 1E) were elevated in the descending thoracic aorta tissues of hypertensive mice, in the descending thoracic aorta samples of SHRs, and in Ang II-treated HUVECs. IF assays revealed increased NAT10 expression in the descending thoracic aorta of hypertensive mice and SHRs compared with that in the control groups (Fig. 1F). Taken together, these results suggest that NAT10 induction mediates ac4C acetylation in complex hypertension progression.

### Overexpression of NAT10 inhibited endothelial dysfunction in hypertension

**Fig. 2:**
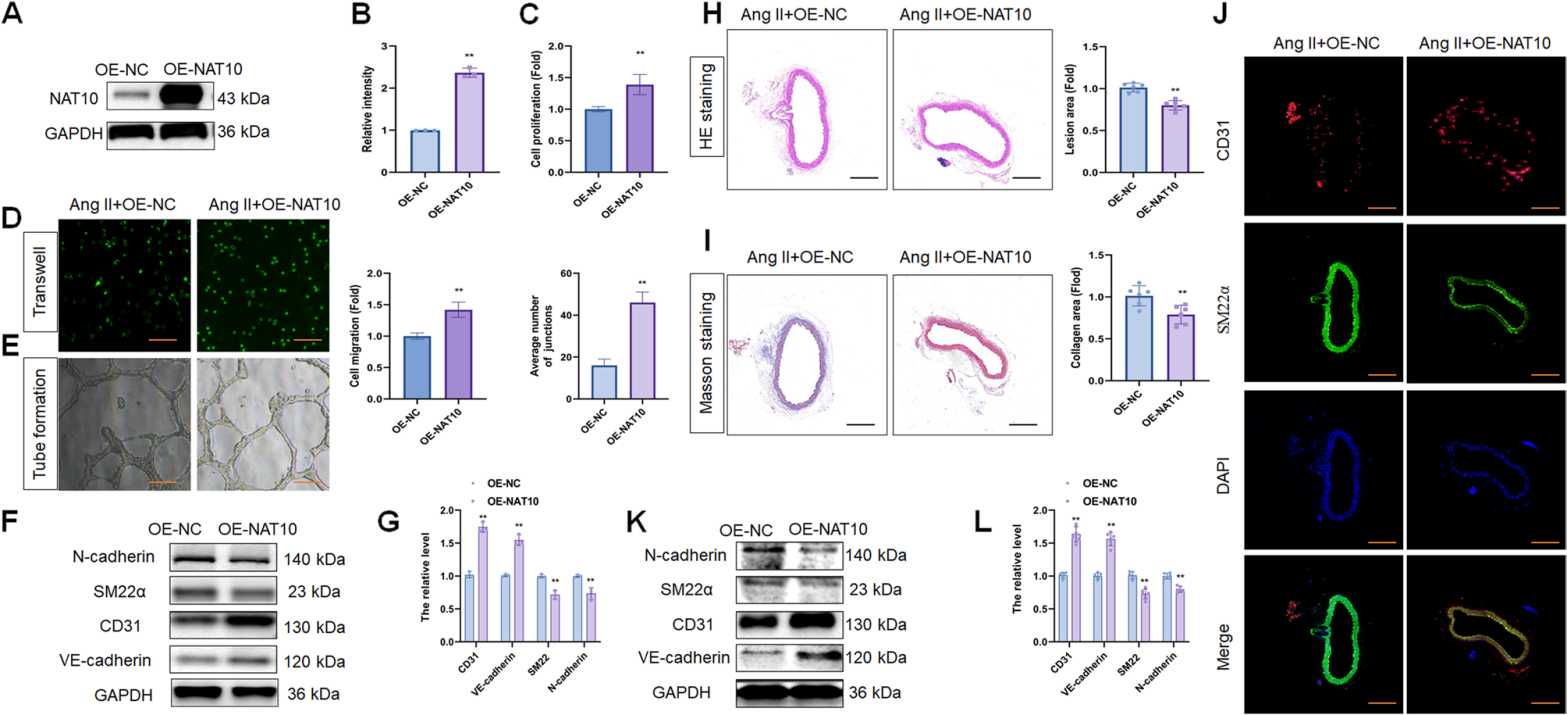
NAT10 overexpression inhibited endothelial dysfunction and EndMT in hypertension. (**A**, **B**) WB assay and the quantitative analysis of NAT10 level in HUVECs after Ang II stimulation (*n* = 3). (**C**) ECs proliferation analysis between OE-NAT10 and OE-NC group after Ang II stimulation (*n* = 3). (**D**) ECs migration analysis between OE-NAT10 and OE-NC group after Ang II stimulation by Transwell migration assay (*n* = 3). Scale bar, 100 μm. (**E**) ECs angiogenesis analysis between OE-NAT10 and OE-NC group after Ang II stimulation by tube formation assay (*n* = 3). Scale bar, 100 μm. (**F**, **G**) WB assay and the quantitative analysis of CD31, VE-cadherin, SM22α and N-cadherin levels in HUVECs after Ang II stimulation (*n* = 3). (**H**) H&E staining of descending thoracic aortic sections and the relative wall thickness of each group (*n* = 6). Scale bar, 100 μm. (**I**) Masson staining of each group and quantitative analysis of the fibrotic area (*n* = 6). Scale bar, 100 μm. (**J**) IF staining of CD31 and SM22α levels in OE-NAT10 and OE-NC group (*n* = 6). Fluorescence in red represents CD31, while fluorescence in green represents SM22α and fluorescence in blue represents DAPI. Scale bar, 100 μm. (**K**, **L**) WB assay of CD31, VE-cadherin, SM22α and N-cadherin levels in OE-NAT10 and OE-NC group (*n* = 6). Data represented as mean ± SD from three independent experiments. **p < 0.01. Statistical tests were performed using unpaired two-tailed Student’s t-test (B-G, L) and Mann–Whitney U test (H, I)

The effects of NAT10 on EndMT and endothelial dysfunction were measured via a gain-of-function assay. The overexpression of NAT10 (OE-NAT10) stimulated NAT10 and ac4C levels (Fig. 2A, B; Fig. S3A), decreased SM22α and N-cadherin levels, and increased CD31 and VE-cadherin levels in Ang II-treated HUVECs (Fig. 2F, G). Moreover, OE-NAT10 increased the proliferation (Fig. 2C), migration (Fig. 2D), and angiogenesis abilities (Fig. 2E) of Ang II-treated HUVECs, indicating that NAT10 overexpression inhibits Ang II-induced endothelial dysfunction and EndMT in HUVECs.

An in vivo assay was conducted to assess the effect of NAT10 on hypertension development. Compared with the control, OE-NAT10 attenuated Ang II-induced SBP (Fig. S2C). Vascular lesions were thinner in the OE-NAT10 group than in the control group after Ang II treatment (Fig. 2H). Compared with that in the control group, Masson’s staining also revealed a decrease in the degree of vascular fibrosis in the OE-NAT10 group after Ang II treatment (Fig. 2I). Additionally, IF revealed that OE-NAT10 mitigated Ang II-mediated decrease in CD31 and increase in SM22α expression (Fig. 2J). Moreover, OE-NAT10 stimulated NAT10 (Fig. S4A, B), CD31, and VE-cadherin expression, which was accompanied by decreases in N-cadherin and SM22α levels compared with those in the control group (Fig. 2K, L). Collagen deposition was measured via the expression of collagen I/III using qPCR and WB. Compared with the control, OE-NAT10 attenuated Ang II-induced increases the mRNA levels of collagen I and III (Fig. S5A). However, OE-NAT10 did not affect Ang II-induced increases the protein levels of collagen I and III (Fig. S5B). Thus, we observed that NAT10 overexpression inhibits EndMT in hypertension, which is partly due to the inhibition of endothelial dysfunction.

### Knockdown of NAT10 induced endothelial dysfunction in hypertension

**Fig. 3:**
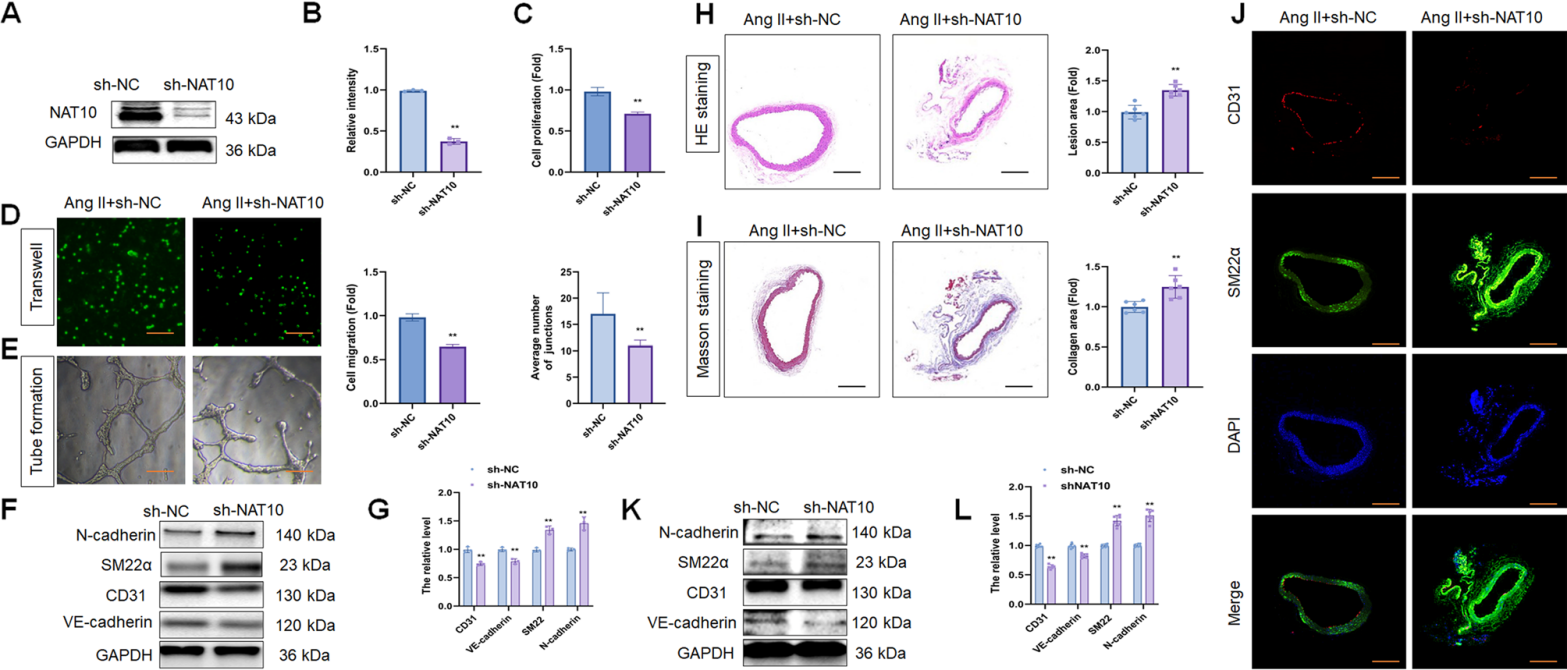
NAT10 depletion induced endothelial dysfunction and EndMT in hypertension. **(A, B) **WB assay and the quantitative analysis of NAT10 level in HUVECs after Ang II stimulation (*n* = 3). **(C)** ECs proliferation analysis between sh-NAT10 and sh-NC group after Ang II stimulation (*n* = 3). **(D)** ECs migration analysis between sh-NAT10 and sh-NC group after Ang II stimulation (*n* = 3). Scale bar, 100 μm. **(E)** ECs angiogenesis analysis between sh-NAT10 and sh-NC group after Ang II stimulation (*n* = 3). Scale bar, 100 μm. **(F, G) **WB assay and the quantitative analysis of CD31, VE-cadherin, SM22α and N-cadherin levels in HUVECs after Ang II stimulation (*n* = 3). **(H)** H&E staining of descending thoracic aortic sections and the relative wall thickness of each group (*n* = 6). Scale bar, 100 μm. **(I) **Masson staining of each group and quantitative analysis of the fibrotic area (*n* = 6). Scale bar, 100 μm. **(J)** IF staining of CD31 and SM22α levels in sh-NAT10 and sh-NC group (*n* = 6). Fluorescence in red represents CD31, while fluorescence in green represents SM22α and fluorescence in blue represents DAPI. Scale bar, 100 μm. **(K, L) **WB assay of CD31, VE-cadherin, SM22α and N-cadherin levels in sh-NAT10 and sh-NC group (*n* = 6). Data represented as mean ± SD from three independent experiments. **p < 0.01. Statistical tests were performed using unpaired two-tailed Student’s t-test (B-G, L) and Mann–Whitney U test (H, I)

NAT10 was knocked down in HUVECs using shRNAs, and sh-NAT10 reduced NAT10 and ac4C levels (Fig. 3A, B; Fig. S3B). Additionally, sh-NAT10 decreased Ang II-treated EC proliferation (Fig. 3C), migration (Fig. 3D), and angiogenesis (Fig. 3E). Furthermore, sh-NAT10 triggered SM22α and N-cadherin expression but decreased CD31 and VE-cadherin levels in Ang II-treated HUVECs (Fig. 3F, G), indicating that NAT10 knockdown increases Ang II-induced endothelial dysfunction and EndMT in HUVECs.

Compared to the control group, sh-NAT10 increased Ang II-induced SBP (Fig. S2D). The vascular thickness was thickening, and the vascular fibrosis formation was increased in the sh-NAT10 group than in the control group after Ang II treatment (Fig. 3H, I). IF results indicated that sh-NAT10 aggravated the decreased CD31 and increased SM22α expression (Fig. 3J). In addition, sh-NAT10 reduced NAT10 (Fig. S4C, D), CD31, and VE-cadherin levels, accompanied by an increase in N-cadherin and SM22α expression (Fig. 3K, L). Compared to the control group, sh-NAT10 increased Ang II-induced the mRNA levels of collagen I and collagen III (Fig. S5C). However, sh-NAT10 did not affect the protein levels of collagen I and III (Fig. S5D). These findings implied that NAT10 inhibition induces EndMT in hypertension, which is partly due to endothelial dysfunction.

### Remodelin induced endothelial dysfunction in hypertension

**Fig. 4:**
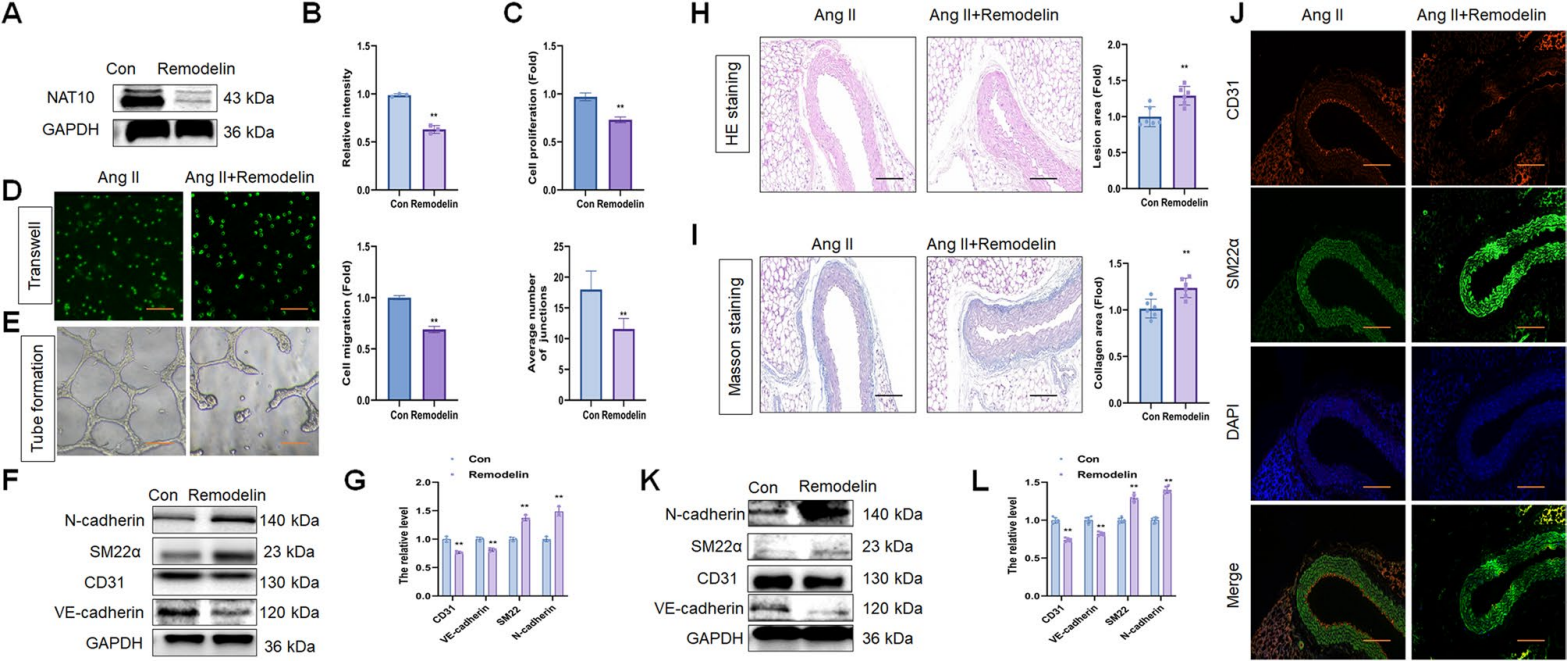
Remodelin induced endothelial dysfunction and EndMT in hypertension. (**A**, **B)** WB assay and the quantitative analysis of NAT10 level in HUVECs after Ang II stimulation (*n* = 3). (**C**-**E)** ECs proliferation, migration and angiogenesis analysis between remodelin and control group after Ang II stimulation (*n* = 3). Scale bar, 100 μm. (**F**, **G**) WB assay and the quantitative analysis of CD31, VE-cadherin, SM22α and N-cadherin levels in HUVECs after Ang II stimulation (*n* = 3). (**H**) H&E staining of descending thoracic aortic sections and the relative wall thickness of remodelin and control group (*n* = 6). Scale bar, 50 μm. (**I)** Masson staining of remodelin and control group and quantitative analysis of the fibrotic area (*n* = 6). Scale bar, 50 μm. (**J)** IF staining of CD31 and SM22α levels in remodelin and control group (*n* = 6). Fluorescence in red represents CD31, while fluorescence in green represents SM22α and fluorescence in blue represents DAPI. Scale bar, 50 μm. (**K**, **L)** WB assay of CD31, VE-cadherin, SM22α and N-cadherin levels in remodelin and control group (*n* = 6). Data represented as mean ± SD from three independent experiments. **p < 0.01. Statistical tests were performed using unpaired two-tailed Student’s t-test (B-G, L) and Mann–Whitney U test (H, I)

Remodelin was used to determine the effect of NAT10 inhibition on endothelial dysfunction and EndMT of HUVECs. The small molecule inhibitor decreased NAT10 and ac4C levels (Fig. 4A, B; Fig. S3C), CD31 and VE-cadherin expression while increasing SM22α and N-cadherin levels (Fig. 4F, G) in Ang II-treated HUVECs. Moreover, remodelin aggravated Ang II-induced damage in HUVECs, including decreased proliferation (Fig. 4C), migration (Fig. 4D), and angiogenesis (Fig. 4E).

Compared to the control group, remodelin treatment increased Ang II-induced SBP (Fig. S2E). The vascular tissue thickened, and the vascular fibrosis formation was increased in the remodelin group compared to the control group after Ang II treatment (Fig. 4H, I). IF results presented that remodelin treatment enhanced the decrease in CD31 and the increase in SM22α expression (Fig. 4J). In addition, remodelin decreased the NAT10 (Fig. S4E, F), CD31, and VE-cadherin levels, accompanied by increased N-cadherin and SM22α expression compared to the control group after Ang II treatment (Fig. 4K, L). Compared to the control group, remodelin treatment increased Ang II-induced the mRNA levels of collagen I and collagen III (Fig. S5E). However, remodelin did not affect the protein levels of collagen I and III (Fig. S5F). Overall, our data indicated that NAT10 inhibitor induces EndMT in hypertension, which is partly due to the endothelial dysfunction.

### NAT10 induced ac4C modification of mitogen-activated protein kinase (MAPK) pathway-related mRNA in Ang II-treated ECs

**Fig. 5:**
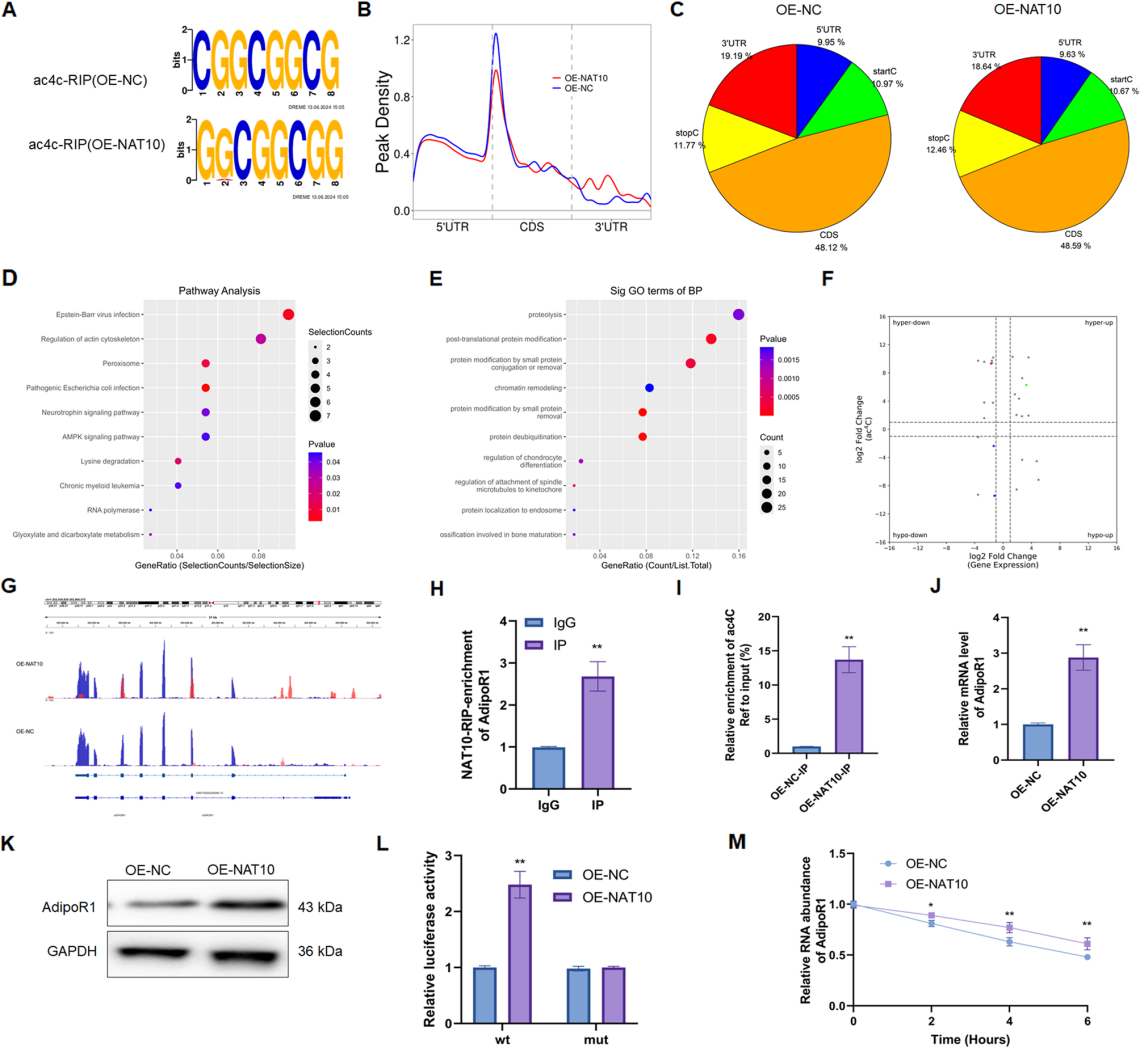
Detection of ac4C modification and selection of NAT10 mediated ac4C modification targets through ac4C-RIP-seq and RNA-seq. (**A)** The ac4C peaks enriched sequence motifs of each group. (**B)** Frequency of ac4C peaks on the mRNAs were exhibited (*n* = 3). (**C)** The pie charts showing the percentage of ac4C peaks on the transcripts. **(D**, **E)** KEGG pathway and GO enrichment analysis exhibited the enriched pathway. **(F) **Combining analysis of acRIP-seq and RNA-seq presented the levels of ac4C acetylation and transcription. (**G)** IGV tracks exhibiting ac4C peaks across AdipoR1 mRNA. (**H)** The interacted connection between NAT10 and AdipoR1 mRNA was detected (*n* = 3). (**I)** The ac4C acetylation of AdipoR1 mRNA of each group were detected (*n* = 3). (**J)** The mRNA level of AdipoR1 in both OE-NC and OE-NAT10 group after Ang II stimulation (*n* = 3). (**K) **The protein expression of AdipoR1 in both OE-NC and OE-NAT10 group after Ang II stimulation (*n* = 3). (**L)** The luciferase reporter assay evaluated the luciferase activities of each group (*n* = 3). (**M)** The effect of NAT10 on AdipoR1 mRNA stability was detected (*n* = 3). Data represented as mean ± SD from three independent experiments. *p < 0.05, **p < 0.01. Statistical tests were performed using unpaired two-tailed Student’s t-test

An ac4C-RIP-seq assay was employed to evaluate the potential effect of NAT10-mediated ac4C acetylation on Ang II-treated HUVECs. The ac4C sequence “CXXCXXCXX” was enriched in the ac4C peaks from the OE-NC and OE-NAT10 groups (Fig. 5A). ac4C acetylation was localized Mainly around the 3’-untranslated region (UTR) and coding sequence (CDS) in both the OE-NC and OE-NAT10 groups (Fig. 5B, C). KEGG analysis revealed that the ac4C acetylation-upregulated genes were enriched in the MAPK signaling pathway after NAT10 overexpression (Fig. 5D). GO analysis revealed that the above genes were enriched in post-translational protein modification (Fig. 5E). Then, the ac4C-RIP-seq and RNA-seq data were pooled to select the downstream targets, which both increased the abundance of ac4C peaks (termed ac4C hyper-peaks) and transcription levels (Fig. 5F). Thus, our data suggest that NAT10-induced targeted ac4C acetylation of mRNAs may be associated with the MAPK pathway in hypertension.

### AdipoR1 is a target of NAT10-induced ac4C acetylation in Ang II-treated ECs

IGV software was used to investigate whether NAT10 altered *AdipoR1* mRNA level via ac4C acetylation. The results revealed increased ac4C modification in the OE-NAT10 group, indicating a potential role of acetylation in enhancing *AdipoR1* mRNA (Fig. 5G). Then, NAT10-mediated ac4C modification of AdipoR1 was further determined by RIP-qPCR, qRT-PCR, and WB analyses. RIP-qPCR data revealed that *AdipoR1* mRNA could bind with NAT10 (Fig. 5H). OE-NAT10 also increased the acetylation of *AdipoR1* mRNA, as shown by ac4CRIP-PCR (Fig. 5I), and elevated the AdipoR1 mRNA and protein levels (Fig. 5J, K) in Ang II-treated ECs.

To verify how ac4C modification modulates *AdipoR1* mRNA expression, WT and mutant (mut) 3’-UTRs of *AdipoR1* reporter gene plasmids were constructed. The luciferase intensity of the WT 3’-UTR of *AdipoR1* increased in the OE-NAT10 group, whereas that of the mut 3’-UTR of *AdipoR1* did not obviously differ (Fig. 5L). Next, the results of the mRNA stability assay revealed that OE-NAT10 increased *AdipoR1* mRNA stabilization in Ang II-treated ECs (Fig. 5M). In conclusion, we deduced that NAT10 increases *AdipoR1* mRNA stability in Ang II-treated ECs via upregulation of ac4C modification.

### Knockdown of AdipoR1 reversed the effect of NAT10 on Ang II-treated ECs

**Fig. 6:**
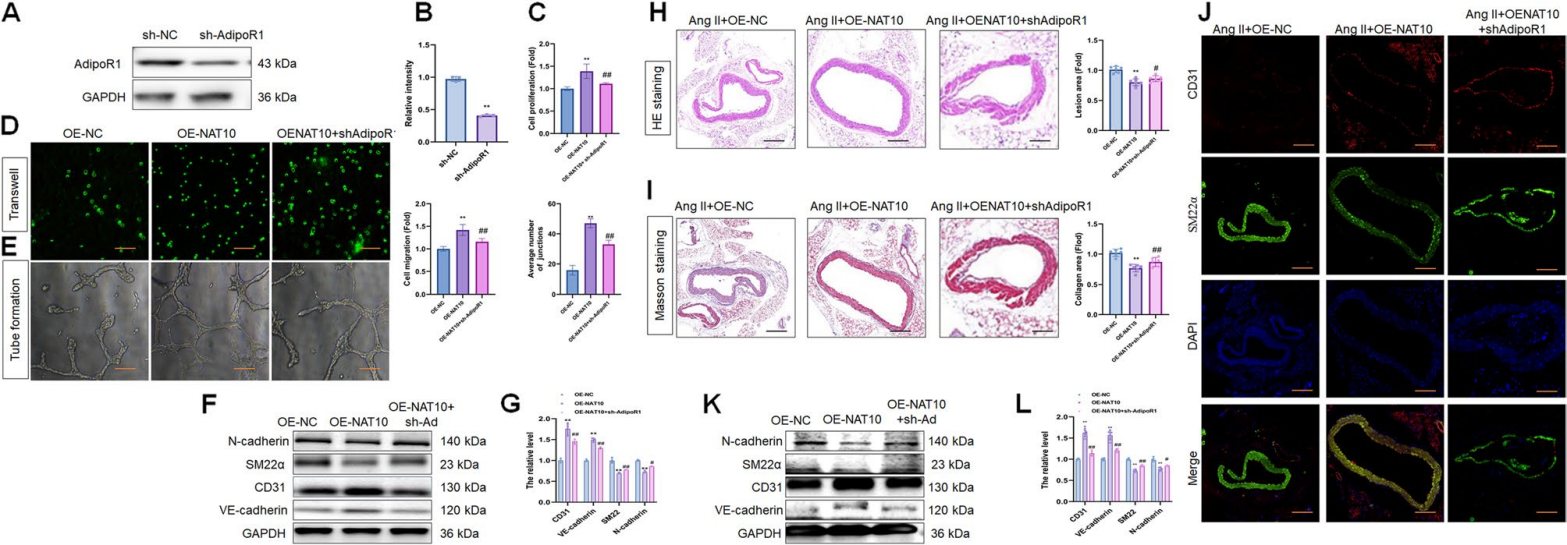
AdipoR1 is a downstream target of NAT10 in Ang II treated ECs. (**A**, **B)** WB assay and the quantitative analysis of AdipoR1 level in HUVECs after Ang II stimulation (*n* = 3). (**C)** ECs proliferation analysis after Ang II stimulation (*n* = 3). (**D)** ECs migration analysis after Ang II stimulation (*n* = 3). Scale bar, 100 μm. (**E**) ECs angiogenesis analysis after Ang II stimulation (*n* = 3). Scale bar, 100 μm. (**F**, **G)** WB assay and the quantitative analysis of CD31, VE-cadherin, SM22α and N-cadherin levels in HUVECs after Ang II stimulation (*n* = 3). (**H)** H&E staining of descending thoracic aortic sections and the relative wall thickness of each group (*n* = 6). Scale bar, 100 μm. (**I)** Masson staining of each group and quantitative analysis of the fibrotic area (*n* = 6). Scale bar, 100 μm. (**J)** IF staining of CD31 and SM22α levels in each group (*n* = 6). Fluorescence in red represents CD31, while fluorescence in green represents SM22α and fluorescence in blue represents DAPI. Scale bar, 100 μm. (**K**, **L)** WB assay of CD31, VE-cadherin, SM22α and N-cadherin levels in each group (*n* = 6). Data represented as mean ± SD from three independent experiments. **p < 0.01, ^#^*p* < 0.05, ^##^*p* < 0.01. Statistical analysis was performed using t-test (unpaired, two-sided) between two groups or one-way ANOVA followed by Tukey’s multiple comparisons test for multiple-group comparisons

To evaluate the role of AdipoR1 in NAT10-mediated EC function, AdipoR1 was knocked down (Fig. 6A, B). Interestingly, sh-AdipoR1 alleviated OE-NAT10 altered Ang II-treated EC capacities (Fig. 6C-E) and the EndMT-related protein expression (Fig. 6F, G). Thus, the current data demonstrated that NAT10 mediates Ang II-treated EC function and EndMT via regulation of AdipoR1 expression in vitro.

Moreover, sh-AdipoR1 mitigated OE-NAT10-attenuated Ang II-induced SBP (Fig. S2F). Compared to the OE-NAT10 group, the vascular thickness was thickened, and the vascular fibrosis formation increased in the sh-AdipoR1 + OE-NAT10 group after Ang II treatment (Fig. 6H, I). According to the IF results, the sh-AdipoR1 + OE-NAT10 group showed decreased CD31 and increased SM22α expression compared to the OE-NAT10 group (Fig. 6J). In addition, sh-AdipoR1 reversed the OE-NAT10-altered EndMT-related protein expression after Ang II treatment (Fig. 6K, L). These data implied that AdipoR1 knockdown reverses NAT10-inhibited EndMT in hypertension, which is partly due to the regulation of endothelial function.

### NAT10-AdipoR1 mediated mitochondrial biogenesis and function through p38 MAPK/PGC-1α signal in Ang II-treated ECs

**Fig. 7:**
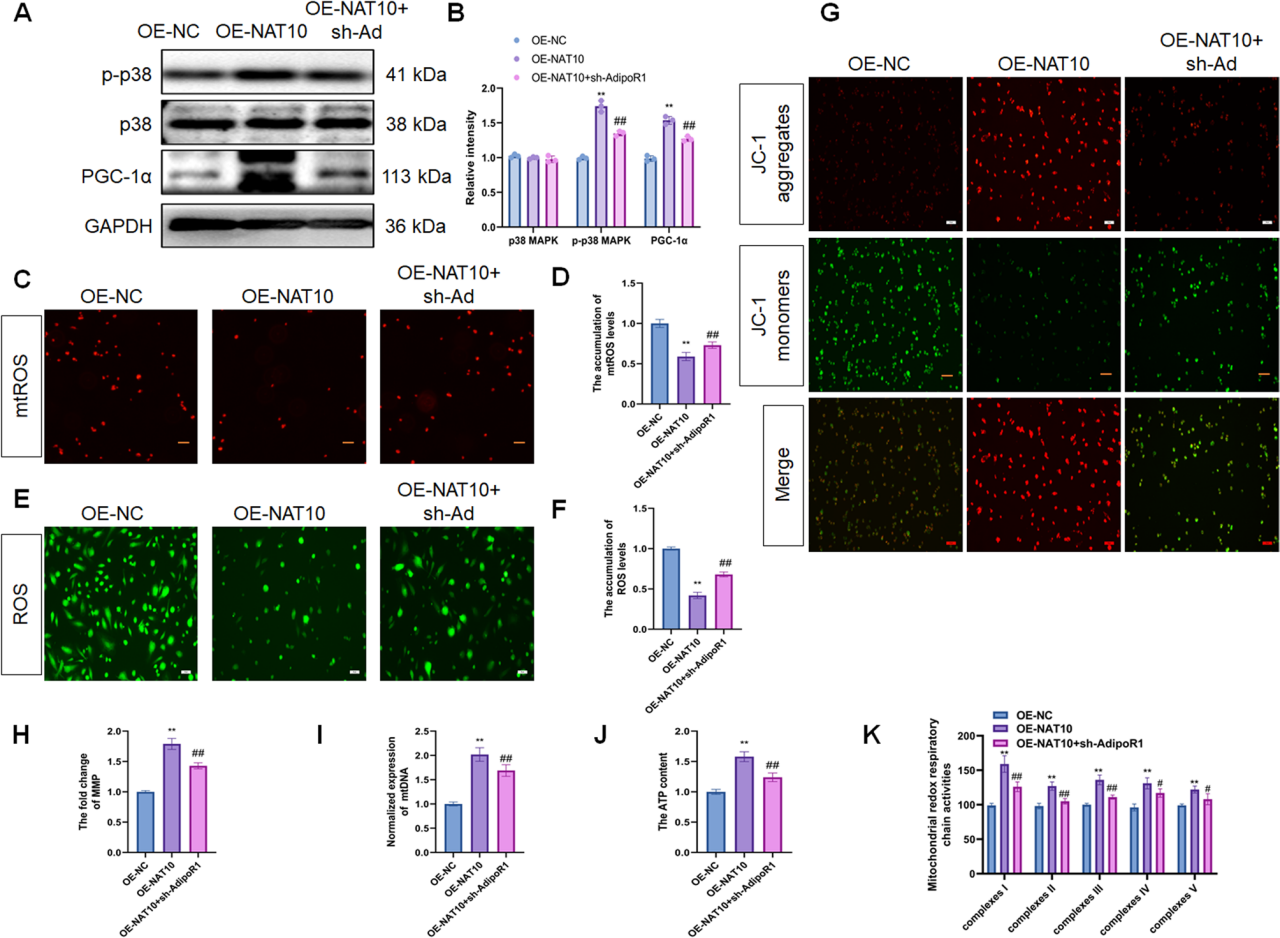
NAT10/AdipoR1 axis mediated mitochondrial biogenesis and function in Ang II treated ECs via the p38 MAPK/PGC-1α pathway. (**A**, **B)** The expression of p38 MAPK and PGC-1α were measured by WB in HUVECs after Ang II stimulation (*n* = 3). The mtROS (**C**, **D**), ROS (**E**, **F**), MMP (**G**, **H**), mtDNA content (**I**), and ATP content (**J**) were detected in HUVECs after Ang II stimulation (*n* = 3). Scale bar, 50 μm. (**K)** The activities of mitochondrial respiratory chain complex were measured in HUVECs after Ang II stimulation (*n* = 3). Data are presented as mean ± SD. **p < 0.01, #*p* < 0.05, ^##^*p* < 0.01. Statistical analysis was performed using one-way ANOVA followed by Tukey’s test

Next, to determine the effect of the NAT10-AdipoR1 axis on mitochondrial biogenesis in Ang II-treated ECs, the expression of the key mitochondrial biogenesis regulators (p38 MAPK and PGC-1α) was detected. The results showed that both p38 MAPK phosphorylation and PGC-1α levels increased in the OE-NAT10 group than in the control group, whereas the levels of these proteins were downregulated in the sh-AdipoR1 + OE-NAT10 group than in the OE-NAT10 group after Ang II treatment (Fig. 7A, B).

Furthermore, to detect the effect of the NAT10-AdipoR1 axis on mitochondrial function in Ang II-treated ECs, we examined ROS, mtROS, and mtDNA content, ATP production, MMP, and the activities of the mitochondrial respiratory chain complex of each group. The intensity of ROS and MitoSOX staining decreased in the OE-NAT10 group compared to the control group and enhanced in the sh-AdipoR1 + OE-NAT10 group than in the OE-NAT10 group after Ang II treatment (Fig. 7C–F). Strikingly, the MMP, mtDNA content, and ATP levels were increased in the OE-NAT10 group than in the control group and decreased in the sh-AdipoR1 + OE-NAT10 group than in the OE-NAT10 group after Ang II treatment (Fig. 7G–J). The activities of the mitochondrial respiratory chain complex presented an increasing trend in the OE-NAT10 group and a decreasing trend in the sh-AdipoR1 + OE-NAT10 group after Ang II treatment (Fig. 7K). These results suggested that the NAT10-AdipoR1 axis facilitates mitochondrial biogenesis and function in Ang II-treated ECs through the p38 MAPK/PGC-1α signal.

## Discussion

Although unique and reversible ac4C modification is involved in different diseases, its role in hypertension remains unclear. The present study revealed the molecular mechanism by which NAT10-mediated ac4C acetylation affects mRNA-related endothelial function in hypertension. Our data revealed that NAT10 and ac4C levels were greater in the hypertensive groups than in the control groups. In vitro assays revealed that NAT10 overexpression inhibits Ang II-induced endothelial dysfunction and EndMT, whereas NAT10 knockdown has the opposite effect. An in vivo assay revealed that NAT10 overexpression reduced vascular thickness and vascular fibrosis formation in hypertensive mice. Moreover, NAT10 overexpression inhibits EndMT in hypertension, which is partly due to the inhibition of endothelial dysfunction. We also observed that NAT10 overexpression inhibits Ang II-induced SBP, which may be related to the regulation of endothelial function. Further investigation revealed that NAT10 inhibits endothelial dysfunction in hypertension through remodeling the ac4C modification of MAPK signaling-related mRNAs, including *AdipoR1*. Moreover, NAT10-induced AdipoR1 expression facilitates mitochondrial biogenesis and function in Ang II-treated ECs via the p38 MAPK/PGC-1α pathway. The current findings highlighted that the NAT10-AdipoR1-PGC-1α axis may be a therapeutic target for hypertension treatment.

Owing to their therapeutic potential in various diseases, the epigenetic modifications of RNA have been broadly explored. Since ac4C modification is the most common type of modified RNA, it has been investigated in various cancers and cardiovascular diseases (Zhang et al. 2024c; Hu et al. 2024; Yan et al. 2023). A recent study revealed that heart apoptosis-associated piRNA regulated TFEC level via ac4C modification during myocardial infarction (Wang et al. 2022). However, the effect of ac4C modification on hypertension has yet to be clarified. To the best of our knowledge, this study provides the first evidence of increased NAT10 and ac4C levels in the descending thoracic aorta tissue of the hypertensive animal model, indicating that NAT10 is involved in hypertension. We found that NAT10 overexpression inhibits EndMT in hypertension, which is partly due to the inhibition of endothelial dysfunction, whereas NAT10 inhibition has the opposite effect. Thus, increased NAT10 expression in EC may serve as an initial adaptive response to hypertension. Moreover, hypertension-stimulated VSMCs may dedifferentiate into a synthetic phenotype, which has hypermigratory and hyperproliferative abilities (Sawma et al. 2022). Intriguingly, NAT10 is expressed not only in ECs, but also in other types of cells, such as VSMCs (Sun et al. 2025). NAT10 expression has been reported to be increased in VSMCs of the atherosclerotic coronary artery, VSMCs of the balloon-injured rat carotid artery, and VSMCs of the wire-injured mouse carotid artery (Yu et al. 2024). Moreover, NAT10 induced VSMC phenotype switching in vivo and in vitro (Yu et al. 2024). Therefore, the increased NAT10 expression in the thoracic aortic tissue of hypertensive mice and SHRs may be partially derived from VSMCs. These findings suggest that NAT10 may participate in hypertension development by regulating both EC and VSMC functions. This finding also revealed that increased NAT10 has different effects on EC and VSMC in complicated hypertension conditions.

NAT10 increases the ac4C acetylation of RNAs, including mRNAs and ncRNAs (Luo et al. 2023). ac4C-RIP-seq and RNA-seq were carried out to detect the effect of NAT10-induced ac4C acetylation on hypertension. An in-depth analysis of ac4C-RIP-seq data revealed that the upregulated ac4C modification of genes were enriched in the MAPK pathway and that dysregulation of the MAPK pathway was associated with hypertension. By combining the ac4C-RIP-seq and RNA-seq data, we identified AdipoR1 as a target of NAT10-induced ac4C acetylation in Ang II-treated ECs. RIP-qPCR data revealed that AdipoR1 mRNA could bind with NAT10. OE-NAT10 also increased the acetylation of AdipoR1 mRNA, as shown by ac4CRIP-PCR. Moreover, OE-NAT10 increased AdipoR1 mRNA and protein levels in Ang II-treated ECs. A previous study revealed that remodeling ac4C acetylation maintains the stability of the 3’-UTR of mRNAs, thereby increasing mRNA translation (Jin et al. 2022). Our luciferase assay results revealed that OE-NAT10 had no obvious effect on the luciferase intensity of the mut-3’-UTR of AdipoR1 but did affect the luciferase intensity of the WT-3’-UTR of AdipoR1. Moreover, NAT10 increased *AdipoR1* mRNA stability in Ang II-treated ECs. Thus, NAT10 induced AdipoR1 expression in Ang II-treated ECs by increasing ac4C acetylation. We subsequently explored whether NAT10 inhibited endothelial dysfunction in hypertension in an AdipoR1-dependent manner. Through molecular and rescue assays, we determined that NAT10 inhibits EndMT in hypertension through AdipoR1 mRNA ac4C acetylation, which is partly due to protection of endothelial function.

AdipoR1 is a key regulator of mitochondrial function; thus, activation of AdiopR1 prevents mitochondrial dysfunction and oxidative injury. Mitochondria are the chief organelles of aerobic respiration in cells. Thus, determining whether NAT10 regulates mitochondrial biogenesis and function in Ang II-treated ECs via AdiopR1 expression is essential. Our results showed that the NAT10-AdipoR1 axis induces mitochondrial biogenesis in Ang II-treated ECs via the p38 MAPK/PGC-1α pathway. Moreover, the NAT10-AdipoR1 axis significantly affects ROS, mtROS, mtDNA content, ATP production, the MMP, and mitochondrial respiratory chain complex activity in Ang II-treated ECs. Collectively, our findings illustrated the role of the NAT10-AdipoR1 axis in hypertension, with a specific focus on mRNA ac4C acetylation-mediated regulation of mitochondrial biogenesis and function in Ang II-treated ECs.

Nevertheless, the present study has several limitations. NAT10 altered mRNA ac4C acetylation, although it has been known to alter the ac4C acetylation of tRNAs and ncRNAs (Liu et al. 2023; Yu et al. 2023). This phenomenon might contribute to the function of NAT10 in hypertension. Since the whole in vitro experiments are conducted using HUVEC, and the adult vascular endothelial cells exhibit anatomical heterogeneity; thus, the use of other type of EC may help us assess the role of NAT10 in hypertension in the future (Tan et al. 2004; Medina-Leyte et al. 2020).The other limitations of this study include the use of only male animals, so the results may not be directly generalizable to female individuals. Gender differences can affect the pathological process of hypertension through hormone regulation (such as the endothelial protective effect of estrogen) or gene expression. The male SHR and C57BL/6 mouse strains were widely employed in previous experimental research targeting hypertension and its comorbid conditions (Kuwahara et al. 2025; Duo et al. 2025; Moraes et al. 2025; Takahashi et al. 2025; Peng et al. 2025). Elevated blood pressure in the male models compare to the age-matched female models (Chen and Meng 1991). In alignment with prior methodologies, our research similarly employed the male SHR and C57BL/6 mice. However, the sexual dimorphism in animals, coupled with sex-specific baseline gene expression may lead to the different outcome in female mice. Therefore, future studies should incorporate both male and female models to facilitate a more comprehensive understanding of the observed phenomena. Moreover, NAT10 may also mediate hypertension through the regulation of VSMC function (Yu et al. 2024). EndMT is a phenotypic transition that shares many markers with VSMC and fibroblasts, making it very difficult to determine whether aortic lesions and collagen accumulation in hypertension are regulated by crosstalk between VSMCs, ECs, and immune cells. The use of inducible Cdh5-Cre with GFP may help us assess the phenotypic role of ECs in the future. Additionally, the potent side effects of targeting NAT10 should not be ignored. Consequently, selecting a therapeutic window that may prolong the protection of EC function but decrease side effects is necessary for hypertension treatment.

## Conclusions

In summary, our study revealed that NAT10-induced ac4C acetylation of *AdipoR1* mRNA inhibits EndMT in hypertension, which is partly due to the inhibition of endothelial dysfunction via increased mitochondrial biogenesis and function. These findings provide in-depth insight into the epigenetics of this disease and may drive the exploration of novel therapeutic strategies for hypertension treatment.

## Supplementary Information

Supplementary Material 1: Figure S1 The weight of animals at the start and end of experiments. Data are presented as mean ± SD. Statistical tests were performed using unpaired two-tailed Student’s t-test.

Supplementary Material 2: Figure S2 The SBP of each group were detected. (A) The SBP between hypertensive mice and the control group (*n* = 6). (B)The SBP between SHRs and WKY group (n = 6). (C) The SBP between OE-NAT10 and OE-NC group (*n* = 6). (D) The SBP between sh-NAT10 and sh-NC group (*n* = 6). (E) The SBP between remodelin and control group (*n* = 6). (F) The SBP between OE-NC group, OE-NAT10 group and OE-NAT10 + shAdipoR1 group (*n* = 6). Data are presented as mean ± SD. **p < 0.01; ^##^*p*<0.01. Statistical analysis was performed using t-test (unpaired, two-sided) between two groups or one-way ANOVA followed by Tukey’s test

Supplementary Material 3: Figure S3 The level of ac4C was detected in HUVECs. (A) The level of ac4C between OE-NAT10 and OE-NC group (*n* = 3). (B) The level of ac4C between sh-NAT10 and sh-NC group (*n* = 3). (C) The level of ac4C between remodelin and control group (*n* = 3). Data are presented as mean ± SD.**p < 0.01. Statistical tests were performed using unpaired two-tailed Student’s t-test

Supplementary Material 4: Figure S4 The level of NAT10 in mice descending thoracic aortic tissues was detected by WB. (A, B) The level of NAT10 between OE-NAT10 and OE-NC group (*n* = 6). (C, D) The level of NAT10 between sh-NAT10 and sh-NC group (*n* = 6). (E, F) The level of NAT10 between remodelin and control group (*n* = 6). Data are presented as mean ± SD.**p < 0.01. Statistical tests were performed using unpaired two-tailed Student’s t-test.

Supplementary Material 5: Figure S5 The relative level of Collagen I and Collagen III of each group were detected. (A, B) The relative mRNA and protein levels of Collagen I and Collagen III between OE-NAT10 and OE-NC group. (C, D) The relative mRNA and protein levels of Collagen I and Collagen III between sh-NAT10 and sh-NC group. (E, F) The relative mRNA and protein levels of Collagen I and Collagen III between Ang II group and Ang II + Remodelin group. Data are presented as mean ± SD. **p< 0.01; ^##^*p* <0.01. Statistical tests were performed using unpaired two-tailed Student’s t-test.

Supplementary Material 6 table s1

## Data Availability

The data analyzed during the current study are available from the corresponding author on reasonable request.
