# Supplementary figures and images for: NAT10 induces N4-acetylcytidine modification of AdipoR1-mediated mitochondrial biogenesis against endothelial-to-mesenchymal transition in hypertension
Source: Mol Med. 2025 Nov 18;31:332. doi: 10.1186/s10020-025-01321-3 (PMC12625234; doi:10.1186/s10020-025-01321-3)

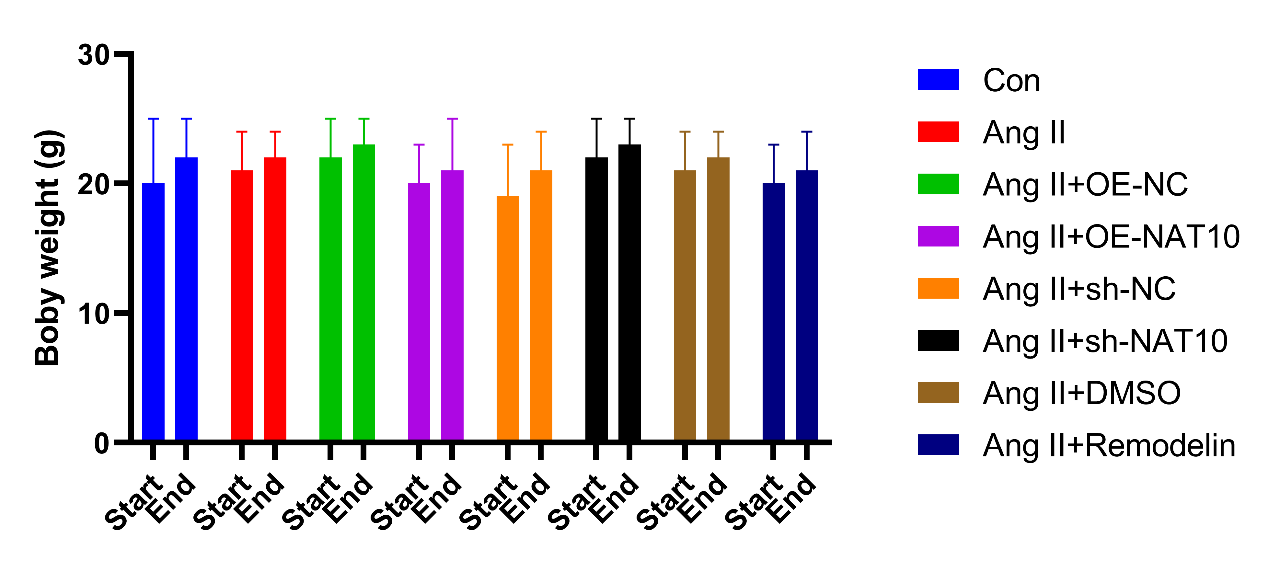

Supplement: Supplementary file 1 — Supplementary Material 1: Figure S1 The weight of animals at the start and end of experiments. Data are presented as mean ± SD. Statistical tests were performed using unpaired two-tailed Student’s t-test. [file 10020_2025_1321_MOESM1_ESM.docx]

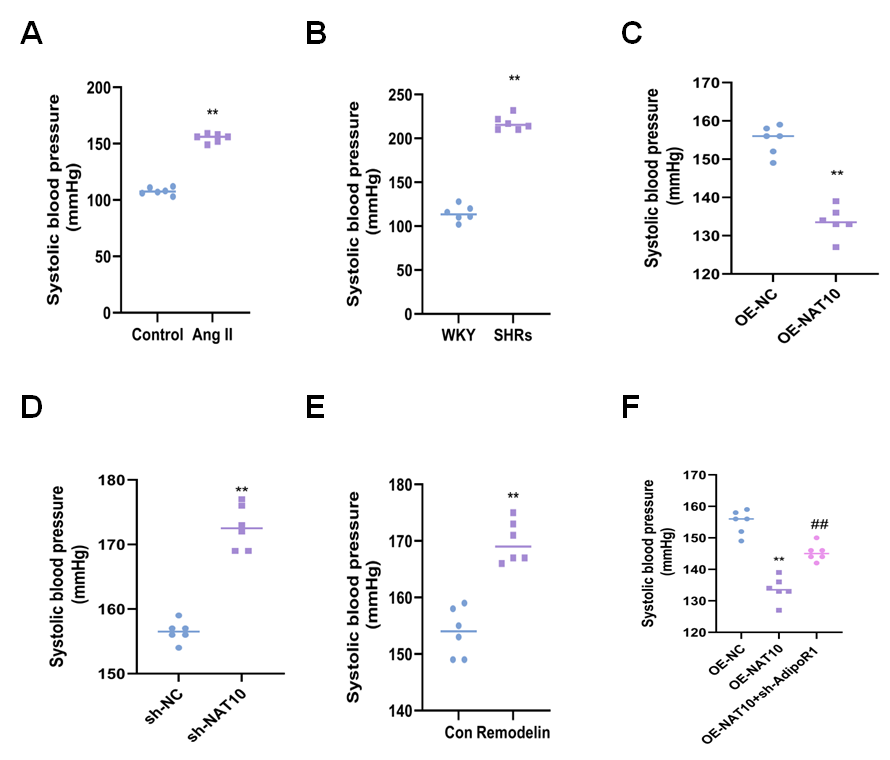

Supplement: Supplementary file 2 — Supplementary Material 2: Figure S2 The SBP of each group were detected. (A) The SBP between hypertensive mice and the control group (n = 6). (B)The SBP between SHRs and WKY group (n = 6). (C) The SBP between OE-NAT10 and OE-NC group (n = 6). (D) The SBP between sh-NAT10 and sh-NC group (n = 6). (E) The SBP between remodelin and control group (n = 6). (F) The SBP between OE-NC group, OE-NAT10 group and OE-NAT10 + shAdipoR1 group (n = 6). Data are presented as mean ± SD. **p < 0.01; ##p<0.01. Statistical analysis was performed using t-test (unpaired, two-sided) between two groups or one-way ANOVA followed by Tukey’s test [file 10020_2025_1321_MOESM2_ESM.tif]

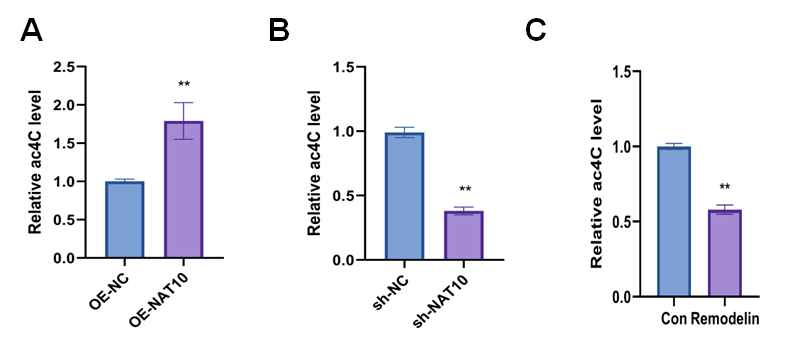

Supplement: Supplementary file 3 — Supplementary Material 3: Figure S3 The level of ac4C was detected in HUVECs. (A) The level of ac4C between OE-NAT10 and OE-NC group (n = 3). (B) The level of ac4C between sh-NAT10 and sh-NC group (n = 3). (C) The level of ac4C between remodelin and control group (n = 3). Data are presented as mean ± SD.**p < 0.01. Statistical tests were performed using unpaired two-tailed Student’s t-test [file 10020_2025_1321_MOESM3_ESM.tif]

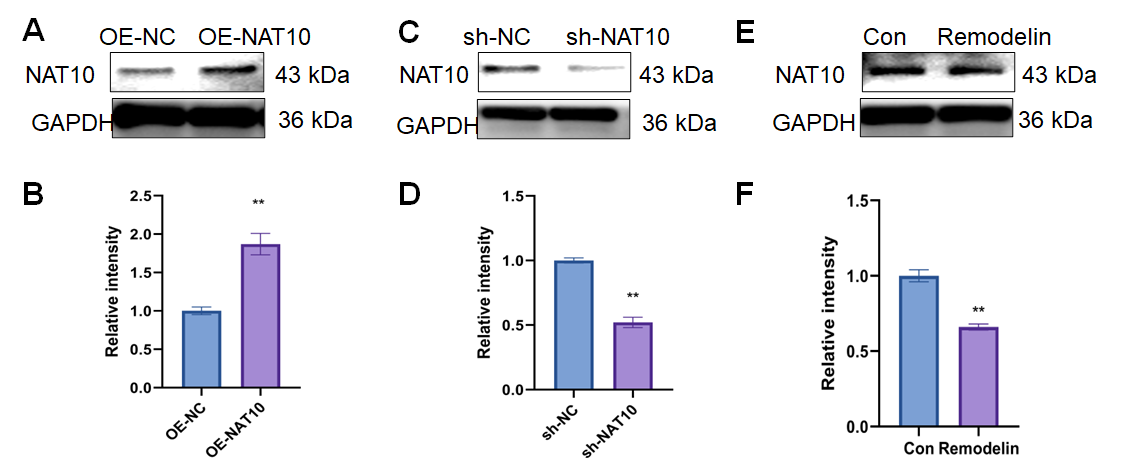

Supplement: Supplementary file 4 — Supplementary Material 4: Figure S4 The level of NAT10 in mice descending thoracic aortic tissues was detected by WB. (A, B) The level of NAT10 between OE-NAT10 and OE-NC group (n = 6). (C, D) The level of NAT10 between sh-NAT10 and sh-NC group (n = 6). (E, F) The level of NAT10 between remodelin and control group (n = 6). Data are presented as mean ± SD.**p < 0.01. Statistical tests were performed using unpaired two-tailed Student’s t-test. [file 10020_2025_1321_MOESM4_ESM.tif]

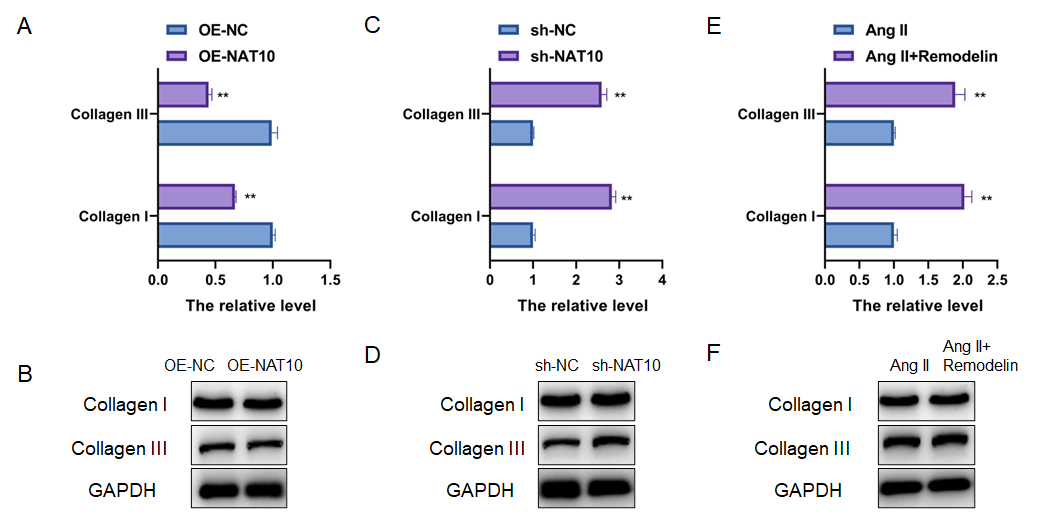

Supplement: Supplementary file 5 — Supplementary Material 5: Figure S5 The relative level of Collagen I and Collagen III of each group were detected. (A, B) The relative mRNA and protein levels of Collagen I and Collagen III between OE-NAT10 and OE-NC group. (C, D) The relative mRNA and protein levels of Collagen I and Collagen III between sh-NAT10 and sh-NC group. (E, F) The relative mRNA and protein levels of Collagen I and Collagen III between Ang II group and Ang II + Remodelin group. Data are presented as mean ± SD. **p< 0.01; ##p <0.01. Statistical tests were performed using unpaired two-tailed Student’s t-test. [file 10020_2025_1321_MOESM5_ESM.tif]
