# Supplementary material for: NAT10 induces N4-acetylcytidine modification of AdipoR1-mediated mitochondrial biogenesis against endothelial-to-mesenchymal transition in hypertension
Source: Mol Med. 2025 Nov 18;31:332. doi: 10.1186/s10020-025-01321-3 (PMC12625234; doi:10.1186/s10020-025-01321-3)
Supplement: Supplementary file 6 — Supplementary Material 6 table s1 [file 10020_2025_1321_MOESM6_ESM.docx]

**The information of the primer sequences used in this experiment.**

Genes Primers Sequence (5′-3′)

m-GAPDH Forward ACCACCATGGAGAAGGCTGG

m-GAPDH Reserve CTCAGTGTAGCCCAGGATGC

m-NAT10 Forward CTGAGCTGGTTGTGATAGATGA

m-NAT10 Reserve CCCTCGTAGCCATTGATAGTAG

m-Collagen I Forward GAGTACTGGATCGACCCTAACCA

m-Collagen I Reserve GACGGCTGAGTAGGGAACACA

m-Collagen III Forward TCCCCTGGAATCTGTGAATC

m-Collagen III Reserve TGAGTCGAATTGGGGAGAAT

H_NAT10 Forward ATGGAGTAGCTGAGCGGCAA

H_NAT10 Reserve CCGAGCCTTCACAGTTGCTT

H_AdipoR1 Forward CTGGCTAAAGGACAACGACTA

H_AdipoR1 Reserve TGTATGAATGCGGAAGATGCT

H_GAPDH Forward GTCTCCTCTGACTTCAACAGCG

H_GAPDH Reserve ACCACCCTGTTGCTGTAGCCAA

**The sequences of NAT10 shRNA for HUVEC**

**No. sequence (5’ - 3’)**

sh-NC TTCTCCGAACGTGTCACGT

sh-NAT10 GGCTGAACTAGTTGTGATTGA

**The sequences of NAT10 shRNA for mice**

**No. sequence (5’ - 3’)**

sh-NC AATTAAAAAAGGATGAAGCAGCAAAGGAATTCTCGAGAATTCCTTTGCTG

CTTCATCCG

sh-NAT10 GATCCGGATGAAGCAGCAAAGGAATTCTCGAGAATTCCTTTGCTGCTTC

ATCCTTTTTT
